# Collaboration under pressure: sustaining One Health research teams in a post-COVID environment

**DOI:** 10.1016/j.onehlt.2025.101298

**Published:** 2025-12-12

**Authors:** Robin B. Gasser

**Affiliations:** Department of Veterinary Biosciences, Melbourne Veterinary School, Faculty of Science, The University of Melbourne, Parkville, VIC 3010, Australia

**Keywords:** One Health, Research teams, Neoliberalism, Managerialism, Academic environment, Collaboration, Sustainable Development Goals (SDGs)

## Abstract

The sustainability of contemporary One Health research increasingly relies on the capacity of universities, research institutes and partner organisations to support collaborative teams. Such teams are indispensable because they integrate diverse expertise, address complex problems and respond rapidly to health crises such as COVID-19. One Health provides a compelling exemplar of collaborative research, uniting human, animal and environmental health to tackle global challenges and emerging infectious diseases. Yet the resilience of such teams is being tested by neoliberal reforms that have reshaped higher education into a competitive marketplace and by managerial practices that prioritise measurable outputs over collegiality, autonomy and disciplinary breadth. The pandemic exposed the fragility of this model: laboratories closed, workloads intensified and financial pressures triggered redundancies, even as collaboration proved essential to global health responses. This Opinion article examines how systemic pressures and interpersonal dynamics intersect to influence the functioning of research teams in a post-COVID environment. It highlights funding precarity, employment insecurity, inequities and political instability as persistent challenges, and explores how performance pressures and competition can affect collaboration. Sustaining research teams requires institutional reforms, inclusive leadership, recognition of diverse contributions and alignment with global frameworks such as the United Nations Sustainable Development Goals (SDGs), so that One Health research and other multidisciplinary endeavours addressing emerging and neglected diseases remain resilient, impactful and socially relevant.

## 1 Introduction

In the twenty-first century, the sustainability of high-quality scholarship in fields such as One Health increasingly relies on the capacity of universities, research institutes and partner organisations to support research teams. Such teams integrate complementary expertise, pursue shared goals and deliver outcomes rarely achievable by individuals working in isolation. Their importance is particularly evident in the biomedical sciences, where research often demands interdisciplinary integration, translational outputs and rapid responses to global health challenges and crises. One Health provides a compelling exemplar of this collaborative model, linking human, animal and environmental health in pursuit of shared objectives that advance global well-being. Before the COVID-19 pandemic, the concept of “high-performance research teams” had already been articulated, highlighting mentorship, equitable workloads and supportive appraisal systems as key enablers of collaboration and productivity in academia [1]. Although this concept originated in nursing, its principles resonate across the biomedical and life sciences, reinforcing the collaborative ethos that underpins One Health research. Ensuring the sustainability of these teams is central to One Health, as their integrative expertise underpins global capacity to prevent, detect and respond to emerging and neglected diseases.

The pandemic, however, exposed the fragility of existing academic structures, particularly in countries where universities had embraced market-driven models privileging efficiency, rankings and external revenues. When international student mobility collapsed, institutions in Australia, the UK, the USA and many other countries faced acute financial crises, triggering redundancies, increased managerial oversight and restricted research opportunities [[2], [3], [4], [5], [6], [7]]. These challenges have not fully abated, with widespread media reports in 2025 of renewed staff cuts and restructures across universities, indicating the continuing financial fragility of the sector. This context also emphasises the risks of neoliberal reliance on tuition income and accelerated reforms that had already eroded collegial governance, constrained academic freedom and entrenched precarious employment [2,3,8]. For early- and mid-career researchers, the crisis revealed both the indispensability of team-based science and the precarity of careers tied to competitive grants with low success rates (often <12 %) [[9], [10], [11]].

Neoliberalism and managerialism provide the structural backdrop to these challenges [[12], [13], [14]]. Since the late twentieth century, universities have been reshaped around market logics, positioning students as consumers, research as a tradable commodity and institutions as corporate actors competing in global rankings [6,15,16]. Managerial cultures have entrenched centralisation, performance audits and accountability metrics [7,14,17]. Together, these forces have shifted leadership from academics to administrators, redefined success around quantifiable outputs and marginalised disciplines misaligned with national priorities. Smaller but globally important areas of One Health, such as parasitology, tropical medicine and epidemiology, illustrate this marginalisation through reductions in discipline-specific courses, diminished representation on grant panels and fewer stable positions (cf. [18,19]). One Health research, particularly in relation to emerging and neglected diseases, provides a clear test case of how disciplinary value is assessed in environments dominated by short-term metrics. It illustrates how collaboration is shaped not only by material resources but also by behavioural dynamics – trust among partners, mobility across borders and recognition of diverse contributions – that determine whether teams can function effectively under pressure [20,21].

Yet COVID-19 also revealed the resilience of research teams. Across the biomedical sciences, consortia rapidly sequenced viral genomes, accelerated vaccine development and integrated epidemiological, clinical, veterinary and social research (e.g., [22,23]). These efforts highlighted the necessity of collaboration across disciplinary and national boundaries, even as academics simultaneously faced disrupted laboratories, online teaching and intensifying job insecurity. Sustaining research teams therefore requires more than goodwill; it demands recognition of the conditions under which collaboration flourishes. Infectious disease research illustrates these tensions most acutely. While such work attracts investment during crises, individual researchers confront unstable contracts and escalating competition. Fields such as tropical medicine and parasitology are particularly vulnerable yet remain essential to One Health research and to achieving several of the Sustainable Development Goals (SDGs), but they often remain sidelined in funding allocations.

This Opinion article revisits the framework proposed earlier [1] in light of post-COVID realities and entrenched neoliberal governance. It argues that while systemic pressures have intensified, they also highlight the need for resilient, well-supported One Health research teams that safeguard academic freedom, value disciplinary diversity and sustain collaborative work in uncertain times.

## 2 The neoliberal university and its fragilities

Research has always been central to the mission of universities, but in recent decades the meaning and value of scholarship have been redefined. Neoliberal reforms have reshaped higher education into a competitive marketplace in which students are framed as consumers, knowledge as a tradable commodity and universities as corporate players competing in global rankings [6,16]. Performance metrics – publication counts, impact factors, grant income and student satisfaction scores – have become dominant indicators of success, driving institutional funding and career progression. What were once developmental appraisal systems now function as institutional tools, rewarding outputs that can be quantified and ranked [17]. This emphasis privileges short-term and measurable research and can undermine scholarship that is collaborative, community-oriented and long-term in trajectory [2,3]. Smaller fields, particularly those misaligned with national priorities, struggle to demonstrate “value” under these regimes [18,19].

Managerialism [14] has intensified these dynamics. Decision-making has become increasingly centralised within executive offices, while academic staff are required to comply with regular performance reviews, workload models and audits [7,24]. Although often justified as accountability mechanisms, such practices can erode collegial governance and academic freedom, creating environments characterised more by mistrust than collaboration. Staff are pressured to document productivity, defend their output and compete with colleagues for shrinking resources. For many early- and mid-career researchers, whose grant success rates often fall to around 10 %, the outcome is more likely to be alienation and burnout than motivation [5,24].

Biomedical sciences illustrate these contradictions vividly. These fields are capital-intensive and heavily dependent on interdisciplinary collaboration, yet they are also among the most exposed to neoliberal dynamics. Governments and funding bodies privilege projects with immediate translational or commercial potential – drug discovery, biotechnology or digital health – while more fundamental or niche disciplines struggle for visibility (cf. [[25], [26], [27]]). Neglected parasitic diseases, for example, are rarely prioritised in national strategies despite their significant impact on vulnerable populations and ecosystems [28,29]. Their apparent exclusion reinforces a paradox: research essential to global health and One Health security is undervalued because its benefits are less tangible and longer term, rather than immediate and commercial. The absence of specialist expertise from national priority lists and funding panels perpetuates this inequity, leaving research teams in these areas to compete in systems that overlook their significance.

The COVID-19 pandemic sharpened these contradictions. It revealed both the indispensability of team science and the fragility of universities reliant on international tuition fees and external grants [5,7]. When campuses closed, researchers were required simultaneously to deliver emergency teaching online, contribute to pandemic responses and absorb unprecedented job cuts [4,6]. For biomedical and One Health teams, the crisis became a lived demonstration of systemic precarity. Far from reversing neoliberal reforms, institutions “doubled down” on performance management and cost-cutting, further entrenching the very conditions that undermine sustainable collaboration [7]. This paradox lies at the heart of the neoliberal university: the qualities most needed to address complex global challenges – collegiality, interdisciplinarity, mentorship and disciplinary diversity – are those eroded by the systems designed to measure and control academic work.

## 3 Competition, pressures and burnout in research teams

Research teams provide the intellectual and practical infrastructure of modern One Health science, yet they remain vulnerable to the systemic pressures and interpersonal dynamics that shape academia more broadly. In neoliberal environments, collaboration can be strained by intense competition for recognition, scarce resources, narrow performance metrics and tensions over authorship or credit [30]. Ambition and confidence – qualities that can stimulate productivity – may in such contexts give rise to tensions that unsettle otherwise effective teams. Large interdisciplinary collaborations, increasingly essential for addressing challenges in the One Health arena, are particularly susceptible as agendas and expectations diverge.

Systemic pressures can erode collegial norms. Neoliberal reforms, expressed through managerialist controls such as performance reviews, workload models and audits, can promote competition over collaboration [7,24]. Within such settings, incivility may take the form of withheld mentorship, limited information sharing or competition for funding and visibility. These behaviours may be influenced by interpersonal dynamics, such as communication styles or expectations, which can place pressure on collaborations across contexts, including with industry [30]. Research fields central to One Health, where ‘status’ is closely linked to high-impact publications and grant income, may be particularly susceptible to hierarchical institutional cultures that can limit inclusivity and reinforce inequality. When resource scarcity combines with these managerial logics and complex dynamics, pressures on trust and collaboration can place team cohesion at risk.

Burnout has become a pervasive threat to collaboration. The abrupt pivot to online teaching, prolonged laboratory closures and intensified workload models since COVID-19 have strained wellbeing across the academic workforce [2,3]. Early- and mid-career researchers face a paradox: their expertise is essential to collaborative projects, yet their career security remains fragile. Leadership styles play a decisive role in determining whether stress translates into resilience or attrition. Leaders who foster inclusive, respectful and transparent cultures buffer their teams from systemic pressures, sustain motivation and protect wellbeing. Conversely, dismissive or domineering leaders may amplify stress, fuelling disillusionment and attrition. The consequences extend beyond individual health; teams disrupted by conflict, incivility or burnout lose competitiveness for grants, face delays in publication and weaken their translational capacity. For underfunded or smaller disciplines, such as parasitology and tropical medicine, the departure of even a few skilled individuals can markedly erode capacity. Building cultures of respect, mutual recognition and psychological safety is therefore an ethical imperative and also a structural necessity for sustaining research teams. Behavioural evidence suggests that cues of working together, reciprocity and shared purpose enhance intrinsic motivation and team cohesion [1,20], reinforcing the principle that collaboration must be cultivated as a social practice rather than assumed as a natural outcome of shared goals.

## 4 Challenges for research teams

The sustainability of research teams working in the One Health arena is shaped as much by institutional and systemic forces as by interpersonal dynamics. Among these pressures is funding precarity. In many countries, success rates for competitive grants have fallen to around 10 %, forcing teams into hyper-competition that consumes enormous time and energy with little prospect of reward (e.g., [[9], [10], [11]]). Funding is also increasingly directed toward narrow national priorities, privileging translational and commercial agendas such as drug discovery, diagnostics and digital health, while fundamental and niche disciplines – such as parasitology or tropical medicine – struggle for visibility [18,[25], [26], [27]]. Neglected parasitic diseases, in particular, are rarely prioritised in funding calls despite their global impact, with recent surveys showing that most investment continues to flow to HIV, tuberculosis and malaria, while NTDs remain underfunded (e.g., [28,29]). This imbalance compromises both disciplinary diversity and the longer term stability of research crucial for global health security.

Funding precarity is mirrored by employment insecurity. Biomedical teams rely heavily on postdoctoral researchers, assistants and technical staff employed on short-term contracts. This creates a “revolving door” of expertise, eroding institutional memory and weakening continuity in long-term projects. Early- and mid-career researchers are particularly vulnerable, often reliant on ‘soft money’ while managing funding, administrative and teaching expectations, conditions that can contribute to uncertainty, reduced morale and, in some cases, exit from academia [[2], [3], [4], [5], [6], [7], [8]]. These vulnerabilities are further compounded by inequities within teams. Gender disparities became acute during the COVID-19 pandemic, with women disproportionately bearing caregiving responsibilities, while international scholars faced travel restrictions, visa insecurity and, in some contexts, exclusionary treatment (e.g., [[31], [32], [33], [34], [35]]). Such inequities weaken creativity and resilience, qualities essential for innovation in biomedical research.

The pandemic also exposed the persistent asymmetries between research teams in the Global North and South. Institutions in the South frequently provided the frontline capacity for surveillance, fieldwork and data collection, yet remained under-represented in the leadership of multinational consortia [[36], [37], [38], [39]]. Analyses of publications reveal a persistent imbalance in credit allocation, with scientists in high-income countries often occupying senior author positions, despite relying on data and expertise generated elsewhere [[36], [37], [38], [39]]. These inequities not only undermine fairness but also weaken scientific capacity by limiting recognition and investment in Southern institutions [39]. Without deliberate measures to rebalance leadership and recognition, the global research ecosystem risks perpetuating epistemic hierarchies that compromise its ability to address transnational health challenges.

Political environments also shape the conditions for team science [[40], [41], [42], [43], [44], [45], [46], [47], [48]]. In some countries, rising populism and authoritarianism have narrowed the space for academic freedom, undermining the trust and openness essential for collaboration [[40], [41], [42], [43], [44]]. In the USA, Trump-era politics accelerated visa restrictions and heightened uncertainty for international researchers, prompting some to consider relocation and contributing to reports of an academic “exodus” [43,44]. In war-affected contexts such as Ukraine, Gaza and Sudan, research infrastructure has been destroyed, staff displaced and international partnerships severed [[40], [41], [42], [43], [44], [45], [46], [47], [48], [49]]. These crises destabilise the global distribution of talent, diminishing capacity in already vulnerable regions and reinforcing inequalities elsewhere. For research teams, this instability is more than a political challenge – it constitutes an existential threat to the continuity of collaboration.

## 5 Toward sustainable team science

If One Health research teams are to thrive in the post-COVID environment, the systems that evaluate and support them need to change. The sustainability of these teams is also fundamental to global health security, as they provide the integrative capacity needed for outbreak detection, pathogen surveillance and cross-sectoral responses to emerging and neglected diseases. Universities and funding bodies continue to privilege individualised outputs – first or last authorships, citation indices, impact factors and grant income – while collaborative contributions such as mentorship, data stewardship, interdisciplinary coordination and community engagement remain undervalued (cf. [50]). Recognition frameworks need to be recalibrated to capture the full spectrum of contributions that make teams function effectively. For smaller or underfunded disciplines central to One Health, where progress often depends on national and international consortia, such recognition is essential. Without it, the systemic incentives for competition and self-promotion will continue to erode collegiality and weaken the very qualities on which high-quality collaboration depends.

The sustainability of research teams also rests on investment in people. Early- and mid-career researchers carry much of the collaborative workload, yet most are exposed to insecure contracts and uncertain career paths. Structured mentoring, leadership development and protection from excessive administrative duties are critical interventions [51]. Teams that actively cultivate diversity – through transparent authorship practices, inclusive decision-making and equitable access to resources – gain resilience and creativity. Leadership style can be decisive: leaders who balance vision with humility can help buffer teams against systemic pressures. By contrast, domineering or hierarchical leadership can accelerate burnout and conflict. Institutions can strengthen resilience by investing in people, mentorship structures, peer-support networks and leadership training that promote inclusive and supportive cultures.

The post-COVID period also calls for a rethinking of how digital technologies are harnessed to sustain collaboration. During the pandemic, virtual platforms enabled teams to continue meeting, sharing data and supervising research across borders. While not a panacea, these tools have reduced costs and expanded participation, offering opportunities to strengthen inclusivity if inequities in access are addressed. Research on virtual learning and team science shows that intentional structures – clear roles, shared goals and reliable communication channels – are critical for effective digital collaboration and open-science practices [52]. Building on these lessons can help teams enhance resilience, particularly for colleagues in regions where travel is restricted or resources are limited.

Research teams can also position themselves within global frameworks that improve visibility and legitimacy. Aligning One Health research with SDGs demonstrates societal relevance and can attract political, collaborative and financial support. Institutions such as the Food and Agriculture Organization (FAO) and World Health Organization (WHO) already promote key agendas (e.g., [53,54]), and research teams that embed their work within this context are better placed to secure resources and recognition. Professional societies also play an important role; they can advocate for marginalised disciplines, foster collaborations and support emerging experts and leaders. By connecting their work to global agendas, while maintaining disciplinary integrity, research teams can navigate the constraints of neoliberal academia and reaffirm their essential role in global health and knowledge creation. Ultimately, sustaining team science is not simply about productivity; it is about protecting academic values of collegiality, creativity, engagement and service, ensuring that research teams remain solid foundations of scientific progress.

## 6 Conclusion

Multidisciplinary research teams are indispensable to the One Health agenda. They provide the collaborative framework and infrastructure needed to tackle complex problems, accelerate discovery and respond to urgent health challenges. Yet their sustainability is threatened by the convergence of neoliberal reforms, managerial oversight and global disruptions. Universities increasingly operate as corporate entities, privileging measurable outputs and short-term gain over collegiality, autonomy and long-term investment. COVID-19 amplified these vulnerabilities, exposing the fragility of funding models, intensifying workloads, expanding casualisation and revealing the uneven distribution of burdens across the workforce [3,5,7].

Within this environment, One Health research teams confront multiple and interlocking challenges: declining grant success rates, short-term contracts, inequities in participation and the corrosive effects of institutional performance management. Competition for scarce resources fuels conflict over respect and recognition, while incivility and ego undermine collegiality. Burnout has become pervasive, particularly among early- and mid-career researchers whose expertise is essential yet whose positions remain precarious. These systemic pressures, together with destabilising forces such as political instability, wars and a persistent North–South divide, compromise the resilience and diversity of the global research workforce.

Despite these challenges, resilience is possible. Teams that cultivate inclusive cultures, distribute credit transparently and protect time for mentorship can manage external stressors. Institutional reforms that recognise collaborative achievements, reduce precarity and streamline administrative demands are essential to sustaining effective collaboration. Professional societies and associations also play a crucial role by advocating for disciplines at risk of marginalisation and aligning them with global agendas such as the SDGs and the Kigali Declaration, launched to support WHO's 2021–30 Road Map for Neglected Tropical Diseases, with commitments from partners including GSK plc, Novartis, Pfizer, Sightsavers and the Wellcome Trust [55,56]. One Health and its associated disciplines, including parasitology, tropical medicine and veterinary sciences, exemplify both vulnerability and adaptability – fields essential to global health yet often underfunded and undervalued. The long-term sustainability of the research teams working in these fields is critical because they enable the cross-sectoral understanding and actions needed to address the underlying causes of ill health that no single discipline or institution can resolve.

Sustaining research teams is, therefore, not simply a matter of productivity but of defending the academic values of collegiality, creativity and service that underpin universities' missions. Without direct action, collaboration risks becoming precarious and unsustainable. With such action, research teams can remain resilient foundations of scientific progress, advancing both biomedical and biotechnological innovation and strengthening global capacity to meet health challenges in an era of uncertainty. Sustaining One Health research, particularly on emerging and neglected parasitic diseases, will be essential to building resilient health systems and achieving equitable global development. More broadly, it will require recognition that research teams are social systems embedded in political and institutional structures, and that their sustainability depends on protecting academic and cultural values, strengthening equitable partnerships and aligning science with the behavioural and policy frameworks needed to achieve key SDGs.

## CRediT authorship contribution statement

**Robin B. Gasser:** Writing – review & editing, Writing – original draft, Conceptualization.

## Declaration of competing interest

The author declares no competing interests.

## Data Availability

No data was used for the research described in the article.
